# Proliferation and osteogenic differentiation of rat BMSCs on a novel Ti/SiC metal matrix nanocomposite modified by friction stir processing

**DOI:** 10.1038/srep38875

**Published:** 2016-12-13

**Authors:** Chenyuan Zhu, Yuting Lv, Chao Qian, Haixin Qian, Ting Jiao, Liqiang Wang, Fuqiang Zhang

**Affiliations:** 1 Department of Prosthodontics, Ninth People’s Hospital, affiliated to Shanghai JiaoTong University School of Medicine, Shanghai Key Laboratory of Stomatology, No. 639, Zhizaoju Road, Shanghai, 200011, PR China; 2 State Key Laboratory of Metal Matrix Composites, Shanghai Jiao Tong University, 800 Dongchuan Road, Shanghai, 200240, PR China

## Abstract

The aims of this study were to fabricate a novel titanium/silicon carbide (Ti/SiC) metal matrix nanocomposite (MMNC) by friction stir processing (FSP) and to investigate its microstructure and mechanical properties. In addition, the adhesion, proliferation and osteogenic differentiation of rat bone marrow stromal cells (BMSCs) on the nanocomposite surface were investigated. The MMNC microstructure was observed by both scanning and transmission electron microscopy. Mechanical properties were characterized by nanoindentation and Vickers hardness testing. Integrin β1 immunofluorescence, cell adhesion, and MTT assays were used to evaluate the effects of the nanocomposite on cell adhesion and proliferation. Osteogenic and angiogenic differentiation were evaluated by alkaline phosphatase (ALP) staining, ALP activity, PCR and osteocalcin immunofluorescence. The observed microstructures and mechanical properties clearly indicated that FSP is a very effective technique for modifying Ti/SiC MMNC to contain uniformly distributed nanoparticles. In the interiors of recrystallized grains, characteristics including twins, fine recrystallized grains, and dislocations formed concurrently. Adhesion, proliferation, and osteogenic and angiogenic differentiation of rat BMSCs were all enhanced on the novel Ti/SiC MMNC surface. In conclusion, nanocomposites modified using FSP technology not only have superior mechanical properties under stress-bearing conditions but also provide improved surface and physicochemical properties for cell attachment and osseointegration.

Due to their biocompatibility and mechanical properties, pure titanium (Ti) and its alloys are widely employed as biomedical metallic materials to replace or repair body parts or functions^1^,^2^,^3^,^4^. Applications for such materials range from bone screws and plates to orthopedic and dental implants^1^,^2^,^3^. However, the poor wear resistance of pure Ti has prevented its application in some medical fields^2^,^5^. The presence of wear debris at the bone-implant stem interface can accelerate bone necrosis^6^,^7^. In addition, the tensile strength of Ti is inadequate for use in orthopedic joints and bone plates and screws^2^. The primary challenge associated with bone implants is the development of materials containing both surface properties that improve cell-substrate interactions and ensure long-term stability of physical and mechanical properties of bioimplants. As such, Ti surface modifications, material composition, and mechanical and tribological properties must be optimized to prolong the life of artificial implants^8^.

Surface properties determine the degree of substrate-tissue interaction integration^9^,^10^. Recently, a variety of methods, such as high-temperature plasma spraying, sol-gel approaches, electrophoretic deposition, pulsed laser deposition, ion-beam deposition and micro-arc oxidation, have been used to modify metal surfaces for dental or orthopedic implants^3^,^11^,^12^,^13^,^14^,^15^,^16^,^17^,^18^,^19^,^20^,^21^. These methods aim to promote implant surface compatibility and to induce bone formation. Bioceramics, particularly hydroxyapatite (HA), have been extensively investigated as potential bone substitutes due to their bioactivity and osteoconductivity. The use of HA coatings on the surfaces of metallic biomaterials allows integration with surrounding bone and facilitates both osteoblast adhesion and long-term function^22^. However, the use of bioceramics in clinical applications is limited due to the poor strength and toughness, weak corrosion resistance, fast dissolution rate and poor interfacial binding force of HA coatings^23^,^24^,^25^. Particles produced by the wear of load-bearing coatings can cause peri-implantitis, which could lead to serious implications, such as implant loosening. Thus, the use of HA in implant devices subjected to stress-bearing conditions is limited. Although HA is advantageous in promoting the proliferation of bone cells through its optimization of the porosity and roughness of implant surfaces, the three-dimensional structure formed at the interface between a substrate and the coating results in a coating adhesion/cohesion strength that is less than the desired value of 35 MPa^3^. The interfacial layer with low mechanical properties will be destroyed and inorganic particles may easily disengaged after implantation^25^. A biomaterial must not only improve surface and physicochemical properties to promote cell attachment and osseointegration but also possess superior mechanical properties for certain load-bearing conditions.

It is reported that metal matrix composites offer increased stiffness, strength and wear resistance over monolithic matrix materials^26^. Enhanced mechanical properties can be obtained when a reinforcing phase is incorporated into a matrix^27^. Nanocomposites based on silicon carbide (SiC) have exhibited enhanced mechanical properties^28^,^29^. Many studies have focused on SiC as a reinforcing particle for the preparation of composite materials. Recent studies of SiC ceramics have indicated that its biocompatibility is comparable to that of HA with respect to its long-term osteogenic properties^30^. Coletti, C *et al*.^31^. demonstrated that the crystalline SiC surface could promote the adhesion, proliferation and differentiation of primary culture osteoblasts. In addition, due to its inertness, SiC is quite promising for overcoming the current drawbacks of biomedical materials and for improving mechanical properties, such as wear and hardness^32^,^33^. Moreover, Si is a crucial element for bone growth and development^34^,^35^.

Nanocomposites with enhanced mechanical properties that could improve bone tissue regeneration are attractive for biomedical applications^36^,^37^. However, it is difficult to create a metal matrix nanocomposite (MMNC) surface while controlling the distribution of nanoscale reinforcements across the metallic substrate using conventional surface treatments. Friction stir processing (FSP) is a surface modification technique based on friction stir welding. The FSP technique has been widely used for the production of surface composites^28^,^38^, the homogenization of metallurgy powders^39^, the microstructural modification of metal matrix composites^40^ and the improvement of physicochemical properties^41^ due to its environmental friendliness and versatility. During FSP, the metallic substrate undergoes intense plastic deformation, leading to effective grain refinement^26^. Misra R.D.K. *et al*.^42^. reported that the nanograined/ultrafine-grained metal substrates produced by the application of extensive plastic deformation could provide superior cell-substrate attachment and biocompatibility. The surface nanocomposites produced by the FSP technique exhibit excellent bonding with substrate, conferring superior mechanical properties^38^. For instance, the pitting and stress corrosion resistances resulting from FSP exceed those of a matrix alloy itself^35^,^43^,^44^. In addition, the yield strength of nanocomposites modified by FSP is enhanced compared with the base material^45^.

Limited information is available regarding the application of FSP technology to biomedical materials. Wang *et al*. claimed that surface strengthening was achieved by the FSP method used to process a Ti-35Nb-2Ta-3Zr β titanium alloy^46^. Unfortunately, few studies have examined the effects of novel MMNC surfaces modified by FSP on cell proliferation and osteogenic differentiation. From the clinical point of view, the new developed biocomposites should have better osteointegration and ability of lowering the incidence of peri-implantitis. We hypothesize that the novel nanograined surface created by FSP promotes cell adhesion and proliferation and leads to effective integration of bioimplants while providing stable physical and mechanical properties. This study aimed to fabricate a novel Ti/SiC MMNC using FSP and to investigate its microstructure and mechanical properties. Additionally, the proliferation and osteogenic differentiation of rat bone marrow stromal cells (BMSCs) on the sample surface were investigated.

## Methods and Materials

### Fabrication of an MMNC by FSP

**Figure 1:**
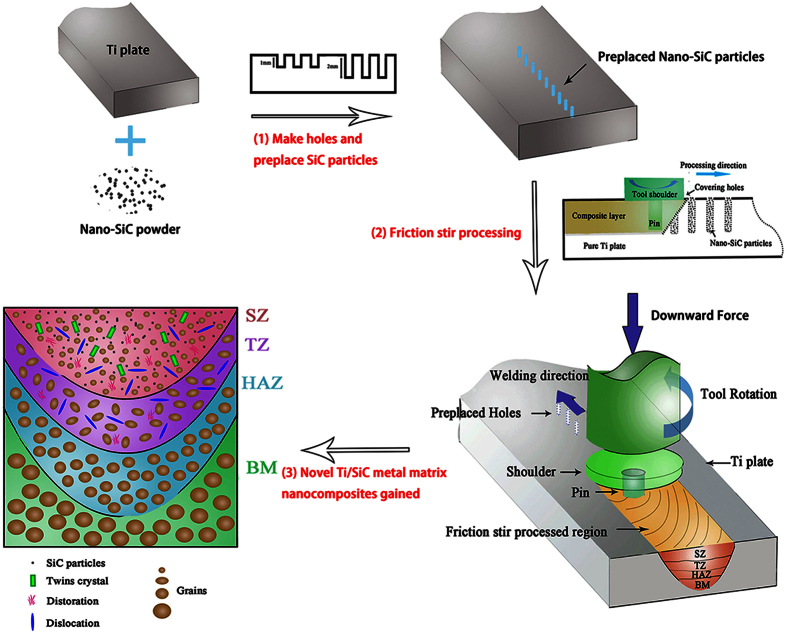
Schematic illustration of the process of producing the FSP-modified Ti/SiC MMNC.

Commercially available SiC powder (99.9% purity with an average particle size of 50 nm; Shanghai Shuitian Materials Technology Co., Ltd., China) and pure Ti plates (Gold Borui Titanium Industry Co., Ltd., China.) were used in this study. The surfaces of the Ti plates were polished and cleaned with an acetone solution. The SiC powder was loaded into holes 1 mm in diameter that were punched into the surface of the work piece. Holes with depths of 1 mm or 2 mm were made in a line at a consistent interval of 2 mm. Accordingly, the Ti base material was denoted as the control group, and the Ti/SiC nanocomposites, which underwent FSP at the different depths of the premade holes, were denoted as the FSP-1 group (1 mm) and the FSP-2 group (2 mm). A constant traverse speed (υ) of 50 mm/min at a rotation rate (ω) of 500 r/min was adopted for the process. During FSP, argon was introduced surrounding both the rotating tool and the surface layers of the FSP zones to prevent high-temperature oxidation of the Ti alloy. FSP was performed using a professional friction stir welding machine with an FSP tool of tungsten steel. The probe was 10 mm in diameter, had a concave shoulder and a 2-mm pin height, and was tilted by 2.5°. The probe was inserted into the work piece for microstructural modification to cover the FSP region. Multiple-pass FSP with 100% cumulative overlap after three passes was implemented for further grain refinement. The schematic in Fig. 1 clearly illustrates the procedural flow used to produce the FSP-modified Ti/SiC MMNC.

Samples with 1 cm*1 cm in size were cut from the stir zone (SZ) of the FSP plate for microstructural examination, mechanical testing and cell experiments.

### Microstructural characterization

For microstructural examination, samples were first extracted from FSP-modified Ti plates using wire electrical discharge machining and then cold-mounted using a self-curing epoxy resin in cylindrical molds. They were then were grounded with 400, 800, 1200 and 2000 SiC abrasive paper. The grounded samples were polished using diamond paste with a particle size of 0.5 μm and then etched with a solution of 6% HNO3+2% HF in water for a few seconds. This sample preparation method meets the ASTM E3–2011 standard and has been widely used in pervious investigations^46^,^47^,^48^. Microstructures were observed using a Quanta 200 microscope as well as scanning electron microscopy (SEM, FEI Company, The Netherlands) and energy dispersive x-ray spectroscopy (EDS, Oxford Inca). To estimate the volumetric ratio of the SiC particles, EDS maps were analyzed using Image-Pro Plus 6.0 software. The microstructures of sample cross-sections were observed by transmission electron microscopy (TEM, JEOL JEM-2100EX).

### Mechanical properties

For mechanical testing, samples were polished to mirror surface. Nanoindentation tests were performed using a NANO Indenter G200 Testing System with a diamond Berkovich tip at continuous loading up to a maximum of 5 mN. Before each unloading process, a 10 s dwelling time was applied at a fixed load. Positions were randomly selected in the center area of each sample, with a distance of 1 mm between two adjacent points. Loading-unloading measurements were performed to determine the nanoindentation depth (h) and elastic modulus. Load and displacement were monitored continuously and recorded to plot a specific curve. The mean values for the nanoindentation depth of each group were compared at five specific loads of 1 mN, 2 mN, 3 mN, 4 mN and 5 mN. Vickers hardness was measured using a microhardness tester (Shanghai Taiming Optical Instrument Co., Ltd.) with a load of 50 g. A group of ten samples was tested.

### Culture and identification of rat BMSCs

Animal experiments were conducted according to the guidelines approved by the Animal Research Committee of the Ninth People’s Hospital affiliated with the Shanghai Jiao Tong University School of Medicine. BMSCs were isolated from the femurs and tibias of six-week-old male Sprague Dawley rats^49^. The cells were cultured in Dulbecco’s modified Eagle’s medium (DMEM) with 10% fetal bovine serum in an incubator with a 5% CO_2_ atmosphere at 37 °C. Non-adherent cells were removed with the first medium change after 24 hours. Cells at passage 2 or 3 were used in subsequent experiments.

Flow cytometry was used to confirm the expression of the surface antigen markers CD29, CD44, CD90, and CD34. A total of 1 * 10^6^ BMSCs at passage 3 were incubated with anti-CD29-fluorescein isothiocyanate (CD29-FITC, eBioscience Inc., San Diego, CA), anti-CD44-phycoerythrin (CD44-PE, eBioscience Inc., San Diego, CA), anti-CD90-allophycocyanin (CD90-APC, eBioscience Inc., San Diego, CA), and anti-CD34-FITC (eBioscience Inc., San Diego, CA) for 1 hour in the dark. The labeled cells were washed, collected, and analyzed using a FACScan flow cytometry system (BD, Franklin Lakes, USA).

### Cell adhesion

For cell experiments, samples were polished with SiC grinding papers of up to 1200 grit^50^. Before sterilization, the samples were cleaned with ethanol in an ultrasonic bath for 5 min. Different samples were plated in 24-well plates. Cells were seeded at a density of 5.0 × 10^4^ cells/well.

After 24 hours, the cells were fixed with 4% paraformaldehyde for 30 min at 4 °C. Next, the samples were sequentially treated with 0.5% Triton X-100 and 3% bovine serum albumin (BSA) at room temperature (RT). To detect the expression of integrin β1, which is a cell adhesion-related protein, a specific primary rabbit-anti-rat antibody targeting integrin β1 (Abcam, Cambridge, MA) was added and incubated with the cells for 8 h at 4 °C^51^. Then, the cells were incubated with an anti-rabbit IgG antibody (Jackson ImmunoResearch Laboratories Inc., USA) for one hour at RT away from light. Optical density was quantitatively analyzed using Image J 1.48 v (National Institutes of Health, USA). The cell cytoskeleton was stained with a FITC-phalloidin antibody (KenGEN BioTECH, China). Nuclei were stained with DAPI for 10 min, and the samples were then observed using a fluorescence microscope (Olympus IX71, Japan).

Cell counts during the initial seeding period (1, 4, and 24 hours) were obtained to represent the cell adhesion properties of the different samples. At each time point, non-adherent cells were removed using a phosphate-buffered saline (PBS) rinse. Adherent cells were detached with a trypsin-EDTA solution (0.25% trypsin with 1 mM EDTA, Gibco). The cells were resuspended to 1 ml and counted using a Z2 Coulter particle count and size analyzer (Beckman Coulter, USA). A group of five samples was examined at each time point.

### Cell proliferation

BMSC proliferation and viability on the samples were assessed by measuring mitochondrial activity using an MTT cell metabolic activity assay. Cells were seeded at a density of 2.0*10^4^ cells/ml onto each sample in a 24-well plate. After culture for 1, 3, 7 and 10 days, MTT solution (5 mg/ml) was added to each well. Approximately four to six hours were required to produce formazan, which was then dissolved in DMSO. The solutions were transferred to a 96-well plate, and the absorbance was measured at 490 nm using an ELX Ultra microplate reader (Bio-Tek, VT, USA). The experiment was repeated three times.

### Alkaline phosphatase (ALP) staining and activity assay

After 7 days of culture, the ALP staining and activity of the BMSCs in each sample were evaluated. For staining, the cells on the samples were fixed with 4% paraformaldehyde for 30 min and incubated with BCIP/NBT kit reagents (Beyotime, China) according to the manufacturer’s instructions. Then, ALP staining was semiquantitatively analyzed using p-nitrophenyl phosphate (Sigma-Aldrich, USA)^49^. Absorbance was measured at 405 nm. After the cells were lysed, total protein content was calculated according to a BSA standard curve method using a Bio-Rad protein assay kit (Bio-Rad, USA) and was measured at 630 nm. ALP activity was determined based on the optical density (OD) value at 405 nm and was normalized to total cellular protein. The experiment was repeated three times.

### Real-time quantitative PCR analysis

BMSCs were seeded at an initial density of 2.0 * 10^4^ cells/ml and cultured in DMEM for 10 days. Total RNA was extracted using a TaKaRa MiniBEST Universal RNA Extraction kit according to the manufacturer’s instructions, and cDNA was synthesized with a TaKaRa PrimeScript 1^st^ Strand cDNA Synthesis kit. The expression levels of genes related to osteogenic and angiogenic differentiation were analyzed using a real-time PCR system (Bio-Rad, USA). The osteogenesis-related genes examined included ALP, osterix (OSX), runt-related transcription factor 2 (RUNX2), osteocalcin (OCN), bone morphogenetic protein 2 (BMP-2), and collagen type 1 (Col 1). The angiogenesis-related genes examined included vascular endothelial growth factor (VEGF), hypoxia-inducible factor-1α (HIF-1α) and angiopoietin-1 (ANG-1). Additionally, the expression of RANKL, an osteoclast differentiation-related gene, was detected by PCR. β-Actin was used as an internal control. The target gene expression levels were calculated using the ΔΔCT method and normalized to the data of the control group. The experiment was repeated three times.

### Immunofluorescence of OCN

BMSCs were cultured for 14 days before the immunofluorescence of OCN was detected in the same manner as that of integrin β1. The initial cell seeding density was 2.0 * 10^4^ cells per well. After localization, the cells were treated with 1% Triton X-100 and 3% BSA, and the samples were incubated with a primary rabbit anti-rat antibody against OCN (Santa Cruz Biotechnology Inc., USA) overnight at 4 °C. Then, the samples were incubated with a red fluorescence-labeled secondary antibody (Jackson ImmunoResearch Laboratories Inc., USA) for another 30 min at RT in the dark. After nuclei were stained with DAPI, the specimens were observed using a fluorescence microscope (Olympus, Japan). Optical density was quantitatively assessed using Image J 1.48 v (National Institutes of Health, USA).

### Statistical analysis

Data were analyzed by ANOVA using SPSS software (version 13.0, SPSS Corporation, USA). The data from each test are presented as the mean ± standard deviation to describe the data distribution. Values of P < 0.05 were considered statistically significant.

## Results

### Microstructures

**Figure 2:**
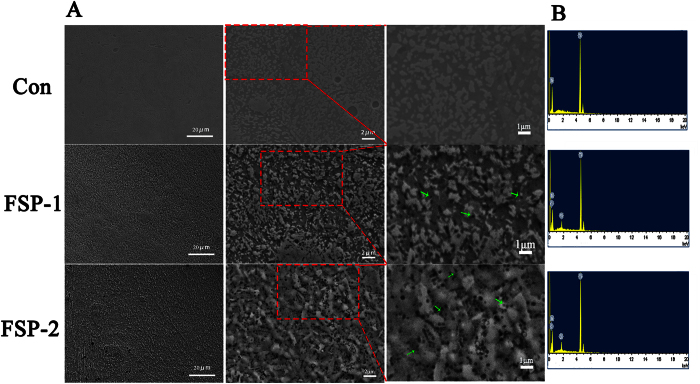
SEM images and EDS spectra of composite surface microstructures. (**A**) SEM images of different magnifications from the three groups. No SiC was detected in the control group. In the FSP-1 and FSP-2 groups, SiC particles were uniformly distributed on the matrix. The green arrows mark the dark SiC particles. (**B**) EDS analysis of the samples confirming the presence of SiC in the FSP-1 and FSP-2 groups.

Based on previous results, the microstructures of the FSP samples often consisted of four primary zones: the base metal (BM), the heat-affected zone (HAZ), the thermomechanically affected zone, and the SZ^38^,^46^. The basin-shaped SZ was used in subsequent cell experiments. No evidence of weld defects was detected. Figure 2A shows SEM images of the top surfaces of the FSP-modified samples. At higher magnification, the SEM images revealed that dark particles were uniformly distributed on the matrix, and these were verified to be SiC particles by EDS (Fig. 2B). Compared to the FSP-1 group, the FSP-2 group exhibited a higher density of SiC particles. The volumetric ratio results based on EDS map analysis showed that the Vol. % of SiC were ~5.1% and ~9.8% in the FSP-1 and FSP-2 groups, respectively. These results suggest that the original quantity of SiC particles had a significant influence on the Vol. % of the SiC particles in the composite.

**Figure 3:**
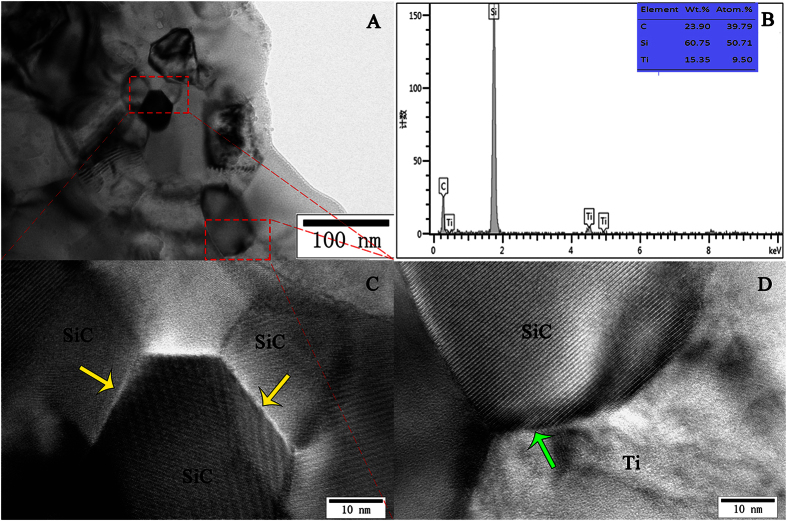
TEM images of interfaces between SiC particles and the Ti matrix. (**A**) The average size of the dispersed SiC particles was approximately 50 nm. (**B**) EDS analysis of the area in (**A**) confirming that SiC particles were present. (**C**) Higher magnification images of those shown in (**A**), revealing the presence of a coherent interface between SiC crystals. The yellow arrows mark the coherent interface. (**D**) Higher magnification images of those shown in (**A**), revealing the presence of a coherent interface between SiC and Ti crystals. The green arrows mark the coherent interface. No chemical reactions occurred between the reinforcing materials and the matrix alloy.

**Figure 4:**
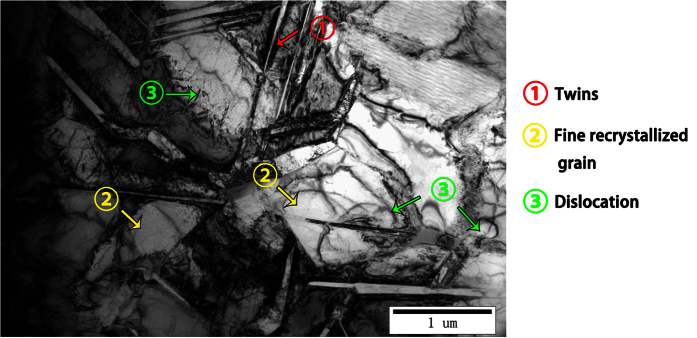
TEM features in the interior of the SZ. The red arrow marks a twin crystal. The yellow arrows mark fine-grained recrystallization. The green arrows mark a dislocation.

The TEM images in Fig. 3A show that the average size of the dispersed SiC particles was approximately 50 nm, which was comparable to that of the added powder, suggesting that the SiC particles were well incorporated into the Ti matrix by way of FSP. Figure 3B verified the SiC crystals by EDS. The higher magnification images in Fig. 3C and D show that the interfaces between SiC and Ti crystals and between two SiC crystals exhibited a coherent relation, which is a type of metallurgical bonding. No chemical reactions were observed between the reinforcing phase and the base material. These findings suggest that a Ti/SiC MMNC was successfully fabricated by FSP. Fig. 4 shows the TEM features in the interior of the SZ. Some twins, fine recrystallization grains, and dislocations were clearly observed, indicating that grains were refined due to plastic deformation and the occurrence of dynamic recrystallization.

### Mechanical properties

**Figure 5:**
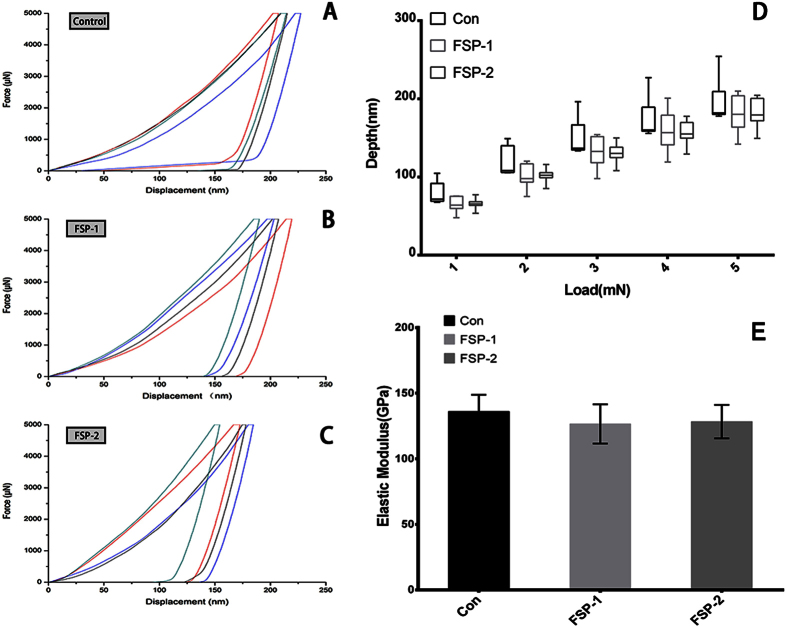
Nanoindentation tests. (**A**) Force-displacement plots for the control group. (**B**) Force-displacement plots for the FSP-1 group. (**C**) Force-displacement plots for the FSP-2 group. (**D**) Load-depth results at five specific loads of 1 mN, 2 mN, 3 mN, 4 mN and 5 mN. (**E**) Elastic modulu**s** results.

**Figure 6:**
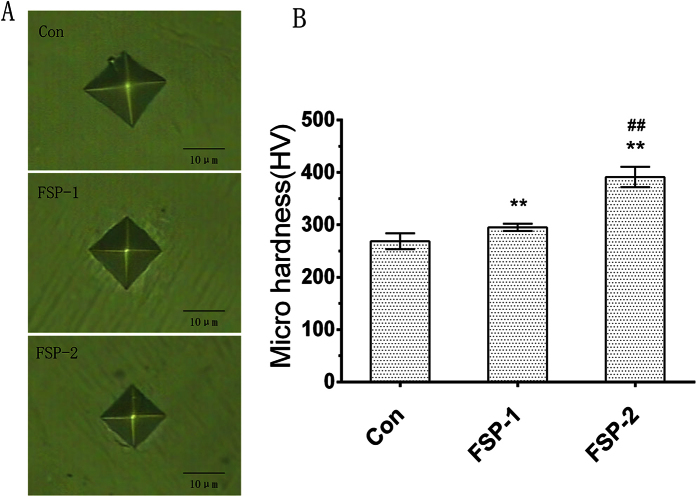
Microhardness tests. (**A**) Region impressed during the Vickers hardness test. (**B**) Microhardness results of each group. (**P < 0.01 compared with the control group; ^##^P < 0.01 compared with the FSP-1 group).

Figure 5A–C illustrate the force-displacement plots for the nanoindentation tests of the three groups. As shown in Fig. 5D, the control group exhibited higher nanoindentation depth values at each loading condition than did the FSP-1 and FSP-2 groups. As shown in Fig. 5E, the elastic modulus decreased with increasing SiC content. The elastic modulus was 136.03 ± 12.81 GPa in the control group and 126.56 ± 15.02 GPa and 128.31 ± 12.78 GPa in the FSP-1 and FSP-2 groups, respectively. While the elastic modulus decreased after the FSP procedure, the Vol. % of the SiC particles had little effect on the elastic modulus. Figure 6A shows the region impressed during the Vickers hardness test. The prismatic area indirectly reflects the microhardness of the sample. As shown in Fig. 6B, the average microhardness was greatest in the FSP-2 group, followed by the FSP-1 and control groups, at 391.13 ± 19.55 HV, 294.94 ± 6.89 HV and 268.53 ± 15.19 HV, respectively. The difference between the FSP groups was statistically significant (P < 0.01). Compared with the control group, the microhardness of the FSP-1 group was increased by 9.83%, and that of the FSP-2 group was increased by 45.7%.

### Identification of rat BMSCs

**Figure 7:**
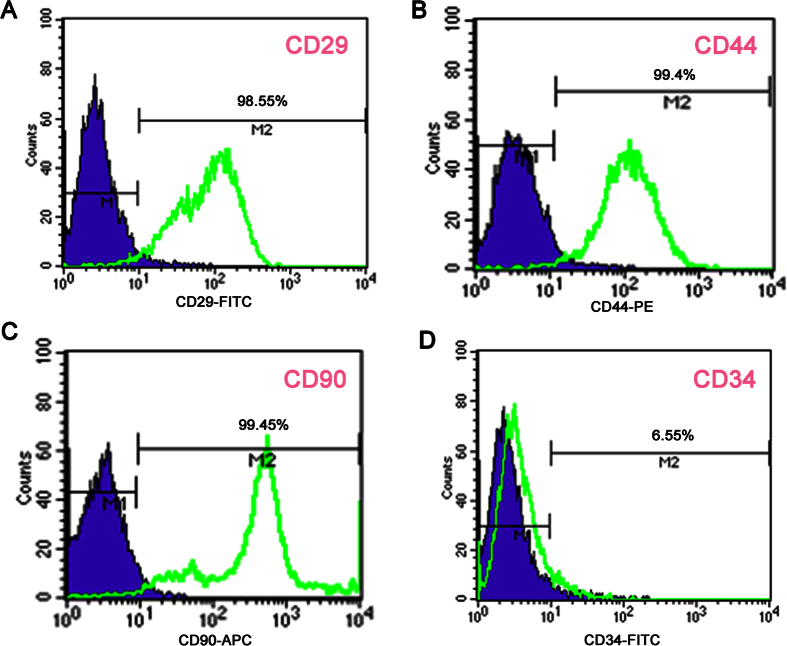
Identification of BMSCs. (**A**) High expression of CD29 (98.55%), (**B**) high expression of CD44 (99.4%), (**C**) high expression of CD90 (99.45%), and (**D**) low expression of CD34 (6.55%).

Analysis by flow cytometry confirmed high expression of CD29 (98.55%, Fig. 7A), CD44 (99.4%, Fig. 7B) and CD90 (99.45%, Fig. 7C), whereas the hematopoietic marker CD34 was rarely detected (6.55%, Fig. 7D). These data confirmed that the cells were BMSCs^52^,^53^.

### Cell adhesion and spreading

**Figure 8:**
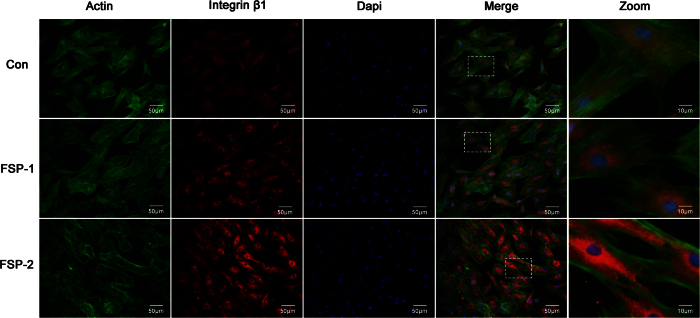
Detection of integrin β1 immunofluorescence and cell spreading after 24 hours of culture. Green represents the actin cytoskeleton of BMSCs stained with FITC-phalloidin; Red represents integrin β1 expression in BMSCs stained with DyLight 549; blue represents the nuclei of BMSCs stained with DAPI.

**Figure 9:**
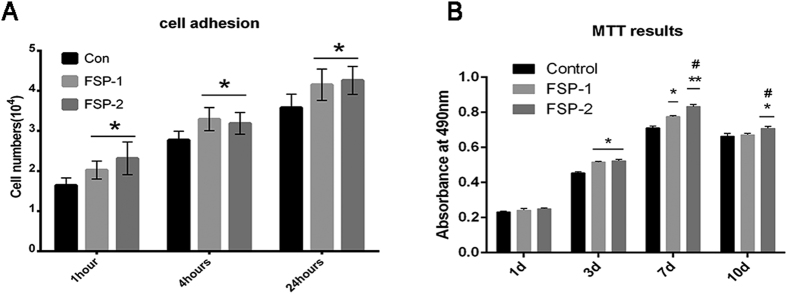
Cell adhesion and MTT assay results. (**A**) Cell numbers at 1 h, 4 h and 24 h in each group. (**B**) Metabolic activity levels of BMSCs on days 1, 3, 7, and 10 of culture, as determined by MTT assay (*P < 0.05, **P < 0.01 compared with the control group; ^#^P < 0.05, ^##^P < 0.01 compared with the FSP-1 group).

As shown in Fig. 8, the BMSCs on each sample showed a multipolar spindle-like morphology and a well-organized cytoskeleton, in agreement with the results of a previous study^54^. The cells extended pseudopodia on all surfaces, consistent with the basic morphology of BMSCs. The surface modification did not impair the spreading of the BMSCs. At 24 hours, integrin β1 was expressed at a higher level in the FSP group than in the control group, while the integrin β1 expression in the FSP-2 group was higher than that in the FSP-1 group (Fig. 8). The relative optical density of integrin β1 expression is shown in Table 1. These results verified the enhancement of rat BMSC adhesion to the Ti/SiC MMNC surface at the protein level. The cell counting results are shown in Fig. 9A. At each time point, more adherent cells were present on the MMNC surface than on the control surface.

### Proliferation or metabolism

The novel Ti/SiC MMNC created by FSP exhibited no cytotoxicity to BMSCs. Figure 9B shows the MTT assay results. No significant differences in total cell metabolic activity were found among the three groups on day 1. However, on day 3, the total cell metabolic activity in the FSP-1 and FSP-2 groups was greater than that in the control group. On days 7 and 10, the cell metabolic activity in the FSP-2 group was significantly greater than that in the FSP-1 and control groups. In addition, the total cell metabolic activity in each group was weaker on day 10 than that on day 7.

### ALP staining and activity

**Figure 10:**
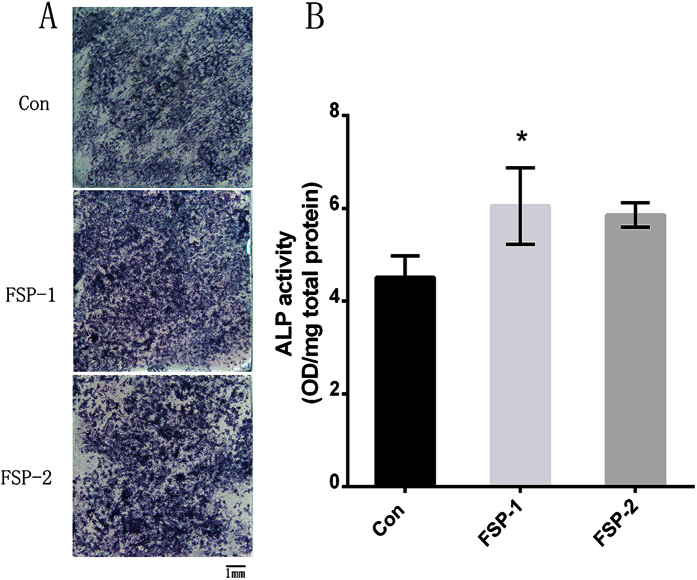
ALP activity assay. (**A**) ALP staining of BMSCs in each sample on day 7 of culture. (**B**) ALP activity of BMSCs in each sample, as determined by p-nitrophenyl phosphate assay on day 7 of culture (*P < 0.05 compared with the control group).

After being cultured for 7 days with DMEM, the BMSCs in the FSP-1 and FSP-2 groups displayed more pronounced ALP-positive staining than did those in the control group (Fig. 10A). Similarly, as shown in Fig. 10B, the quantitative results revealed that the ALP activity was increased in the FSP groups compared with the control group, and there was a significant difference between the FSP-1 and control groups (P < 0.05).

### Real-time quantitative PCR analysis

**Figure 11:**
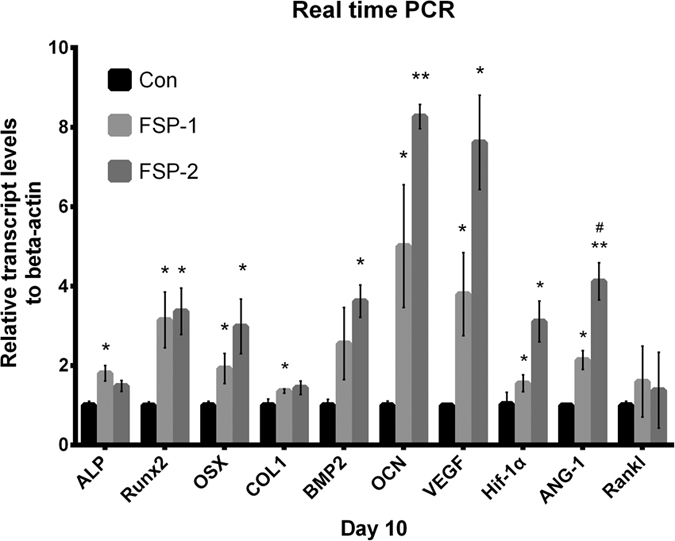
Expression levels of osteogenic and angiogenic differentiation marker genes. Expression of the ALP, OSX, RUNX2, OCN, BMP-2, Col 1, VEGF, HIF-1α, ANG-1, and RANKL genes on day 10 assayed by real-time PCR (*P < 0.05, **P < 0.01 compared with the control group; ^#^P < 0.05, ^##^P < 0.01 compared with the FSP-1 group).

As shown in Fig. 11, real-time PCR was applied to detect the expression of both osteogenesis-related and angiogenesis-related genes. Compared with the base material, the FSP surfaces led to the upregulation of all the examined mRNAs, particularly RUNX2, OSX, and OCN, as well as the angiogenic factors VEGF, HIF-1α and ANG-1. These results suggest that the Ti/SiC MMNC surface modified by FSP exerted robust positive effects on the later stages of osteogenic differentiation and the stages of vascularization. The OSX, OCN, VEGF, HIF-1α and ANG-1 genes were expressed at a higher level in the FSP-2 group than in the FSP-1 group. The upregulation of these mRNAs illustrated an enhancement in osteogenic and angiogenic differentiation due to the different surface and physicochemical properties. However, no differences were detected in the expression of RANKL.

### Immunofluorescence of OCN

**Figure 12:**
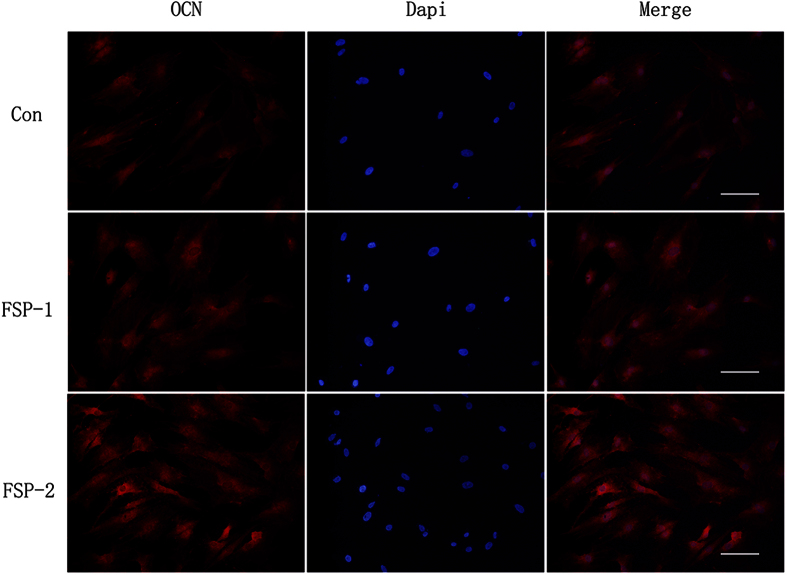
Detection of OCN immunofluorescence. The expression of OCN was detected by fluorescence microscopy. Red represents OCN expression in BMSCs stained with DyLight 549; blue represents the nuclei of BMSCs stained with DAPI. Scale bar, 50 μm.

Immunofluorescence was adopted to further detect the expression of OCN at the protein level. According to the fluorescence intensity shown in Fig. 12, the cells seeded on the FSP surfaces expressed higher levels of OCN than did the cells seeded on the base material. In addition, the cells in the FSP-2 group expressed a higher level of OCN than did the cells in the FSP-1 group. The relative optical density of OCN expression is shown in Table 2. Thus, the Ti/SiC MMNC surface modified by FSP was confirmed to enhance osteogenic differentiation at the protein level.

## Discussion

In our study, when the constant travel speed was set at 50 mm/min and the rotation speed was set at 500 rpm during FSP, the Ti matrix was refined and SiC particles were well incorporated into the Ti matrix due to the extensive plastic deformation and frictional heating produced by the process of FSP^55^. After completing up to three passes, the SiC particles became uniformly distributed on the Ti matrix (Fig. 2), and the matrix was significantly refined by the resulting dynamic recovery and recrystallization (Fig. 3). In addition, interfaces between the SiC and Ti crystals and between two SiC crystals both exhibited a coherent relation (Fig. 3C and D). Furthermore, no evidence of defects or porosity was detected. Therefore, in this work, a Ti/SiC MMNC was successfully fabricated.

Compared to the control material, the MMNC exhibited a lower elastic modulus and greater microhardness. The fine recrystallized grains of the matrix and the homogenization of the reinforcing particles within the SZ contributed to the reduced elastic modulus. Nanocomposites produced by FSP have very small grains; thus, a large volume fraction of atoms reside in the grain boundaries^56^. Atoms in grain boundaries have greater spacing compared with internal atoms, and the elastic modulus is a measure of bonding between atoms^57^. Therefore, material that possesses a more refined grain will have a lower elastic modulus. This may be the reason why the nanocomposite created in our study has a low elastic modulus. Additionally, there are two main explanations for the enhanced microhardness. First, the Vol. % of SiC in the FSP-1 group was ~5.1%, whereas the Vol. % of SiC in the FSP-2 group was ~9.8%. This increase in the Vol. % may have led to increased microhardness because of the ability of the particles to prevent slip deformation of the matrix. Nanometer SiC particles also act as reinforcements, which can also increase the hardness of the nanocomposite. Second, as the temperature in the center of the SZ exceeds the transformation temperature of the β phase during plastic deformation and friction, the material undergoes recrystallization^46^. Under the influence of high temperature, the grain refinement and phase transition in the matrix positively affected the hardness of the material^26^. Our TEM results indicate that some twins and high-density dislocations also formed due to the plastic deformation caused by FSP. Similar results have been obtained in previous studies^58^,^59^,^60^. Fujii *et al*.^59^. attributed the increased hardness of pure Ti submitted to FSP to the presence of fine grains and high-density dislocations. In our study, fine recrystallization grains were obtained after FSP, as indicated by the yellow arrows in Fig. 4, which ultimately increased the strength of the specimen. The FSP-modified Ti/SiC MMNC surface exhibited clear improvements in mechanical properties.

Both 316 L stainless steel and Co-Cr-based alloys have been used for surgical implants. Elements such as Ni, Cr and Co are released from these alloys due to the corrosion they undergo in the physiological environment^61^. The toxic effects of released Ni, Co and Cr elements have been reported in previous studies^61^,^62^. In addition, compared with 316 L stainless steel and Co-Cr-based alloys, Ti and its alloys have a lower elastic modulus, better corrosion resistance, enhanced biocompatibility and greater specific strength^63^,^64^. Therefore, Ti and its alloys have great advantages for use as implant biomaterials. It has been reported that the Al and V ions released from Ti6Al4V alloy are associated with long-term health problems. Vanadium is also toxic both in its elemental state and as an oxide (V_2_O_5_)^64^. Thus, pure Ti is the best choice for use as an implant. However, pure Ti has poor shear strength, making it less desirable for bone screws^65^. Debris generated by the wear of pure Ti can lead to inflammatory reactions, causing pain and loosening of implants^66^. Therefore, the current work aimed to improve the wear-related properties of Ti by causing severe plastic deformation of the material surface in addition to the inclusion of nanometer-sized SiC particles on the surface. The addition of SiC particles improved the mechanical properties of the nanocomposite. Furthermore, SiC has good biocompatibility that is comparable to HA with respect to its long-term osteogenic properties^31^. Our results also indicate that the nanocomposite has superior mechanical and physicochemical properties.

Although FSP technology is well established, few previous studies have investigated the adhesion, proliferation and osteogenic differentiation of rat BMSCs on MMNC surfaces modified by FSP. The nanoscale surface topography produced by FSP modification with SiC particles is significantly beneficial for cell adhesion, an event required for subsequent cellular functions, including osteogenic differentiation^51^,^67^. Both measures of integrin β1 expression levels and cell counting results demonstrated that cell adhesion was enhanced on the surface of the FSP-modified material. Successful adhesion has a crucial role in forming a physical link between integrin receptors and extracellular matrix proteins, which participate in cell signal transduction in response to external stimuli^51^. The increased expression of integrin improved the stimulation of cell signal transduction to some extent, which could promote cell proliferation. In addition, the focal adhesion kinase/extracellular signal-regulated kinase signaling pathway is strongly related to osteogenic differentiation activity in cells^51^,^68^. The MTT assay results confirmed that the metabolic activity of the cells in the FSP groups was greater than that of the cells in the control group at 3, 7, and 10 days. Cell metabolic activity at least partially represents cell proliferation rate, and the observed increase in cellular metabolism was most likely due to an increase in cell number. Thus, the MTT assay results indicated that the Ti/SiC MMNC surface exerted a positive effect on cell proliferation.

Differences in surface chemistry might underlie the enhancements in cell adhesion and proliferation that have been observed on substrates containing SiC^31^. In the present study, these enhancements were likely due to the incorporation of SiC into the Ti base material at the nanoscale level by means of FSP. Elemental Si is reported to have a positive effect on osteoblast proliferation^28^,^69^. In addition, SiC has a large energy bandgap (>1.8 eV), which may reduce electronic interactions (i.e., charge exchange) between cell adhesion proteins and SiC surfaces. Reduced electronic interactions are beneficial for cell adhesion^31^. The enhancement of cell adhesion through the formation of nanocomposites has been widely reported^55^,^70^,^71^. Nanomaterials have exhibited the promising capability of stimulating cell function and enhancing tissue regeneration^36^,^37^,^72^,^73^. In addition, nanomaterials can possess biomimetic features and unique surface properties, including unique physicochemical, mechanical, and biological properties^74^. Nanophase composites have higher proportions of surface atoms and surface electron delocalization^55^,^75^. In this study, fabricated MMNC surfaces with different surface and physicochemical properties were created by varying the SiC content. Both initial protein interactions and subsequent cell adhesion characteristics were affected by these altered properties.

BMSCs have been a focus of stem cell-based tissue engineering researchers over the last decade. BMSCs are multipotent stem cells that can differentiate into bone, cartilage and adipose cells as well as many other cell types^76^,^77^. Stem cell differentiation into different lineages is accompanied by significant changes in cell morphology, and cell shape has an effect on various biological processes of MSCs, such as proliferation and differentiation^78^. McBeath *et al*.^79^,^80^. observed that MSCs that adhere and spread are more likely to undergo osteogenesis, while rounded cells without good spread commonly become adipocytes. Moreover, Engler *et al*.^81^. reported that BMSCs undergo osteogenic differentiation when cultured on a stiff matrix. To gain insight into the osteogenic effect of the created Ti/SiC MMNC in the absence of extra inductive additives, the osteogenic potential of rat BMSCs was assessed based on ALP activity, real-time PCR and OCN immunofluorescence. Increased ALP-positive staining and increased ALP activity indicated the augmented osteogenic potential of the BMSCs cultured on the FSP-modified samples. The osteogenic gene markers ALP, RUNX2, OSX, Col 1, BMP-2, and OCN were analyzed to assess the osteogenic abilities of the BMSCs at the gene level. ALP is an early marker of osteogenesis, and its upregulation indicates enhanced phosphate metabolism. As an osteoblast transcription activator, RUNX2 is involved in the regulation of gene expression during the process of osteogenic differentiation. OSX plays an important role in osteogenic maturation^49^. Col I, which provides the structural framework for inorganic molecule deposition, affects the biomechanical strength of bone tissue^82^. BMP-2, a member of the TGF-β family, is crucial for the activation and regulation of bone formation^83^. The expression level of OCN reflects the degree of the deposition and mineralization of cells, which regulates the later stages of osteogenic differentiation^49^. The expression of RUNX2 activates the osteogenic process and stimulates OSX, Col-1, OCN and BMP-2 expression, which is involved in the maturation and stabilization of osteoblasts. OCN immunofluorescence verified the improvement in the osteogenic function of the studied BMSCs at the protein level. These results indicate the advantageous effects of the novel nanocomposite surface on the osteogenic differentiation of BMSCs. In addition to the chemical composition of biomaterials, their wettability, crystallinity, surface topography and porous structure are able to regulate the attachment, spread, migration, morphology and function of cells^84^. Slight variations in these properties can produce quite different results. For example, in the current study, the specific texture found on the Ti/SiC MMNC facilitated cell adhesion and increased integrin expression. The upregulated integrin expression was positively related to the number of contact spots existing between cells and matrix. These contact spots improved cellular transduction in response to external stimuli. Notably, the focal adhesion kinase/extracellular signal-regulated kinase signaling pathway is related to osteogenic differentiation^51^,^68^. Additionally, as shown in Fig. 11, OSX and OCN expression significantly increased in the FSP-2 group relative to the FSP-1 group. Notably, the smaller SiC nanoparticles that were present in the FSP-2 group possessed a large specific surface area. These positive effects indicate the importance of the volumetric content of SiC in differentiation. Furthermore, metallic materials possessing nanometer-sized grains contain surfaces that differ from conventional polycrystalline materials because of the large proportion of grain boundaries with high free energy^42^. Angela Carvalho^78^ demonstrated that mesenchymal stem cells (MSCs) can undergo osteogenic differentiation solely in response to microtopographic stimuli, and different geometric shapes triggered different levels of osteogenesis. In the present study, BMSCs seeded on Ti/SiC MMNC in the absence of extra inductive additives were confirmed to have underwent osteogenic differentiation, providing strong evidence that positive cellular responses occur on nanostructured metal substrates.

Angiogenesis and osteogenesis are tightly coupled during bone development and regeneration^85^. BMSCs have the potential to promote angiogenesis, which makes them an ideal cell type for the engineering of vascularized tissue. The present study also analyzed the effect of novel Ti/SiC MMNC on the expression of angiogenic factors by BMSCs. The expression levels of the angiogenesis-related markers VEGF, ANG-1 and HIF-1α were detected. It is well known that these angiogenic factors could simultaneously promote osteogenesis and angiogenesis^83^,^86^. VEGF is a key angiogenic factor for enhancing blood vessel formation that effectively regulates biological activity^87^. ANG-1 is essential for the growth and interactions of endothelial cells with pericytes during the later stages of blood vessel formation^75^. VEGF is an important target gene of HIF-1α, which directly regulates the expression of VEGF at the gene level and promotes vessel formation. RT-PCR results showed that the Ti/SiC MMNC stimulated the expression of VEGF, ANG-1 and HIF-1α, which in turn promoted osteogenesis of BMSCs. With regard to the expression of RANKL, the Ti/SiC MMNC showed no effect on the osteoclastogenic-related factors of BMSCs.

Generally speaking, FSP is an effective surface modification technique for joining a metal matrix with a reinforcing phase to form a nanocomposite surface. In the current study, the reinforcement provided by SiC particles enhanced the mechanical properties of the SZ. The nanophase surface produced by modification with SiC nanoparticles within the SZ significantly improved cell adhesion and proliferation. Both osteogenesis and angiogenesis were enhanced on this modified surface, indicating the prospects of this technique for tissue-engineering applications.

However, in this paper, the elastic modulus were 126.56 ± 15.02 GPa in the FSP-1 group and 128.31 ± 12.78 GPa in the FSP-2 group. These values are still higher than the values of natural bone tissue. An ongoing challenge in the field is to obtain biomaterial with an elastic modulus that is close to that of human bone. Furthermore, how this MMNC promotes responses in rat BMSCs requires further elucidation. In addition to the SZ, future studies should focus on the HAZ and the TZ, and other reinforcements, such as Ag and TiO, should also be evaluated for the ability to improve the mechanical and physicochemical properties of the nanocomposite.

## Conclusions

Based on assessments of microstructure and microhardness, FSP is a very effective technique for modifying Ti/SiC MMNC surfaces to contain uniformly distributed particles at the nanoscale level. The adhesion, proliferation and osteogenic differentiation of rat BMSCs cultured on a novel modified Ti/SiC MMNC surface were enhanced. This nanocomposite exhibited not only superior mechanical properties suitable for stress-bearing applications but also improved surface and physicochemical properties for cell attachment and osseointegration.

## Additional Information

**How to cite this article**: Zhu, C. *et al*. Proliferation and osteogenic differentiation of rat BMSCs on a novel Ti/SiC metal matrix nanocomposite modified by friction stir processing. *Sci. Rep.* **6**, 38875; doi: 10.1038/srep38875 (2016).

**Publisher's note:** Springer Nature remains neutral with regard to jurisdictional claims in published maps and institutional affiliations.

## Figures and Tables

**Table 1:** Relative optical density of integrin β1 expression.

Groups	IOD/pixel(Mean ± SD)	P value
Control	0.025 ± 0.0030	<0.001
FSP-1	(0.034 ± 0.0021)*	
FSP-2	(0.043 ± 0.0045)*,#	

IOD/pixel represents the ratio of the integrated density and the area. (^*^P < 0.05 compared with the control group, ^#^P < 0.05 compared with the FSP-1 group).

**Table 2:** Relative density of OCN expression.

Groups	IOD/pixel(Mean ± SD)	P value
Control	0.036 ± 0.0041	<0.001
FSP-1	(0.041 ± 0.0053)	
FSP-2	(0.058 ± 0.0092)*,#	

IOD/pixel represents the ratio of the integrated density and the area. (^*^P < 0.05 compared with the control group, ^#^P < 0.05 compared with the FSP-1 group).
